# Antimetastatic Potential of Rhodomyrtone on Human Chondrosarcoma SW1353 Cells

**DOI:** 10.1155/2020/8180261

**Published:** 2020-07-27

**Authors:** Malatee Tayeh, Ramida Watanapokasin

**Affiliations:** ^1^Department of Medical Technology, School of Allied Health Sciences, Walailak University, Nakhon Si Thammarat 80160, Thailand; ^2^Department of Biochemistry, Faculty of Medicine, Srinakharinwirot University, Bangkok 10110, Thailand

## Abstract

Chondrosarcoma is primary bone cancer, with the forceful capacity to cause local invasion and distant metastasis, and has a poor prognosis. Cancer metastasis is a complication of most cancers; it is one of the leading causes of cancer-related death. Rhodomyrtone is a pure compound that has been shown to induce apoptosis and antimetastasis in skin cancer. However, the inhibitory effect of rhodomyrtone on human chondrosarcoma cell metastasis is largely unknown. Effect of rhodomyrtone on cell viability in SW1353 cell was determined by MTT assay. Antimigration, anti-invasion, and antiadhesion were carried out to investigate the antimetastatic potential of rhodomyrtone on SW1353 cells. Gelatin zymography was performed to determine matrix metalloproteinase-2 (MMP-2) and MMP-9 activities. The effect of rhodomyrtone on the underlying mechanisms was performed by Western blot analysis. The results demonstrated that rhodomyrtone reduced cell viability of SW1353 cells at the low concentration (<3 *μ*g/mL); cell viability was >80%. Rhodomyrtone at the subcytotoxic concentrations (0.5, 1.5, and 3 *μ*g/mL) significantly inhibited cell migration, invasion, and adhesion of SW1353 cells in a dose-dependent fashion. Protein expression of integrin *αv*, integrin *β*3, and the downstream migratory proteins including focal adhesion kinase (FAK) and the phosphorylation of serine/threonine AKT, Ras, RhoA, Rac1, and Cdc42 were inhibited after treatment with rhodomyrtone. Moreover, we found that rhodomyrtone decreased the protein level of MMP-2 and MMP-9 as well as the enzyme activity in SW1353 cells. Meanwhile, tissue inhibitor of metalloproteinase-1 (TIMP-1) and TIMP-2 expression was increased in a dose-dependent fashion. Besides, rhodomyrtone dramatically inhibited the expression of growth factor receptor-bound protein-2 (GRB2) and the phosphorylated form of extracellular signal regulation kinase1/2 (ERK1/2) and c-Jun N-terminal kinase1/2 (JNK1/2). These results indicated that rhodomyrtone inhibited SW1353 cell migration, invasion, and metastasis by suppressing integrin *αvβ*3/FAK/AKT/small Rho GTPases pathway as well as downregulation of MMP-2/9 via ERK and JNK signal inhibition. These findings indicate that rhodomyrtone possessed the antimetastasis activity that may be used for antimetastasis therapy in the future.

## 1. Introduction

Chondrosarcoma, a malignant bone cancer, is the most frequent type of primary bone cancer [1]. Conventional chondrosarcoma is the most frequent all chondrosarcoma (80–90%). It can arise in any bone, but the most common sites are femur, humerus, tibia, pelvis, and ribs. It is more commonly seen in adults aged 20 to 60 years [2]. The metastatic capacity of conventional chondrosarcoma correlates with histological grading, about 15% of all metastatic disease-related deaths occur more than 5 years after diagnosis [3]. High-grade chondrosarcomas comprise grades II and III chondrosarcoma that are very aggressive and frequently metastasize to the lung [4]. They are highly insensitive to radiotherapy or chemotherapy; thereby the surgical resection remains the primary method of therapy [5]. Cancer metastasis is the principal cause of mortality in cancer patients. Cancer metastasis consists of multiple steps involving the spread of tumor cells from the original site, migration and invasion through the basement membrane, survival in the circulatory system, and proliferation into a secondary site [6]. Integrins are primary receptors for cellular adhesion, mediating dynamic cell-cell and cell-extracellular matrix interactions [7]. They are a heterodimeric transmembrane glycoprotein which formed from different combinations of 18 *α*-subunits and 8 *β*-subunits that can associate to form 24 unique integrin heterodimers [8]. They have been implicated in the metastasis of ovarian pancreatic, lung, bladder, osteosarcoma, and chondrosarcomas [9–14]. Overexpression of *α*2*β*1, *αvβ*1, *αvβ*3, *β*1, and *β*1 integrin in human chondrosarcoma is associated with tumor progression, metastasis, and poor prognosis [5, 8, 15, 16]. Integrins initiate transmembrane signaling through activation of the downstream effectors such as FAK, PI3K/AKT, and Rho GTPases family for cancer cell migration [8, 17–19]. Small Rho GTPases including RhoA, Rac1, and Cdc42 are well known to regulate cell migration [20], and overexpression of these molecules in human cancer correlates with cancer progression [21]. There are many reports demonstrating that overexpression of RhoA associated with various cancers' progression such as testicular cancer, breast cancer, colon, lung cancers, and head and neck squamous cell carcinoma [22–24]. Rac1 is overexpressed in various cancers such as testicular cancer [22], breast cancer [23], and leukemia [25]. Furthermore, overexpression of Cdc42 has been reported to be in several human cancers, such as breast, non-small cell lung cancer, and melanoma [23, 26, 27]. Besides, several studies indicated that the expression of matrix metalloproteinases (MMPs) is regulated by integrin signaling [28, 29]. MMP-2 and MMP-9 are the important proteases involved in cancer cell migration, spreading, invasion, and metastasis [30]. They are highly expressed in various malignant tumors, and activation of these enzymes enables the degradation of ECM by cancer cells allowing their access to the vasculature as well as migration and invasion into the target organ and development of cancer metastasis [31–33].

*Rhodomyrtus tomentosa*, a medicinal plant, is native to Southern and Southeast Asia, from India, Sri Lanka, east to southern China, Hong Kong, Taiwan, Philippines, Thailand, south to Malaysia and Sulawesi and USA [34]. It is used as a traditional medicine for the treatment of many diseases such as anti-inflammation, antidiarrheal, laxative, carminative, and urinary tract infections [35, 36]. Rhodomyrtone is a pure compound isolated from the leaves of this medicinal plant species. It exhibited antibacterial activities, immunomodulatory activity, antiproliferation, delayed wound closure, and induced apoptosis in HaCaT cells [37–42]. Our recent studies demonstrated that rhodomyrtone induced apoptosis in skin cancer [43]. It was also reported as antimetastasis of human skin cancer cells [44]. However, the effect of rhodomyrtone on the metastasis in chondrosarcoma cells is largely unknown. In this study, molecular mechanisms underlying the antimetastasis effect of rhodomyrtone in SW1353 cells were investigated in vitro to determine the potential of rhodomyrtone as a novel chemotherapeutic drug.

## 2. Materials and Methods

### 2.1. Chemicals and Reagents

Rhodomyrtone was provided by Dr. Wilawan Mahabusarakum, Department of Chemistry, Faculty of Science, Prince of Songkla University. Dimethyl sulfoxide (DMSO), ethylenediaminetetraacetic acid (EDTA), gelatin A, *β*-mercaptoethanol, and 3-(4,5-dimethylthiazol-2-yl)-2,5-diphenyl-2H-tetrazolium bromide (MTT) were purchased from Sigma-Aldrich (St. Louis, MO, USA). Matrigel Matrix was obtained from Corning Incorporated (Bedford, MA, USA). Coomassie Brilliant Blue R-250 and protein assay kit were purchased from Bio-Rad Labs (Hercules, CA, USA). Rabbit monoclonal anti-MMP-2, -MMP-9, -TIMP-1, -TIMP-2, -RAS, -GRB2, ERK1/2, p-ERK1/2, p38, p-p38, JNK1/2, pJNK1/2, FAK, AKT, pAKT (Ser473), pAKT (Thr308), NF-*κ*B, RhoA, RAC1, CDC42, Integrin *αv*, integrin *β*3 and *β*-actin were purchased from Cell Signaling Technology (Danvers, MA, USA).

### 2.2. Cell Culture

Chondrosarcoma cell line SW1353 (ATCC, Manassas, VA, USA) initiated from a primary grade II chondrosarcoma of the right humerus from a 72-year-old Caucasian female. SW1353 was cultured in DMEM (Gibco, Grand Island, NY, USA) supplemented with 10% fetal bovine serum (FBS) (GE, Healthcare, Life Science, Little Chalfont, UK) and antibiotic (100 U/mL of penicillin and 100 *μ*g/mL of streptomycin) (GE, Healthcare, Life Science, Little Chalfont, UK). The cells were incubated at 37°C in a humidified atmosphere of 5% (v/v) CO_2_.

### 2.3. Cell Viability Assay

Cell viability was analyzed using MTT assay as previously described [43]. Briefly, 3 × 10^3^ cells of SW1353 were seeded in 96-well plates and incubated with or without rhodomyrtone (0–5 *μ*g/mL) for 0, 6, 12, and 24 h. DMSO was added for dissolving the formazan crystals. Absorbance was detected at a wavelength of 570 nm by an Epoch™ Microplate spectrophotometer. Percentages of cell viability (%) were determined by comparing with the untreated control.

### 2.4. Apoptotic Cells Assay

SW1353 cells were seeded in a 6-well plate at a density of 1.5 × 10^5^ cells/well. After treatment with or without rhodomyrtone (0–5 *μ*g/mL) for 24 h, the treated cells were washed with phosphate-buffered saline (PBS), fixed with 3.7% paraformaldehyde, and stained with Hoechst 33342 for 15 min. The nuclear morphological changes were observed under a fluorescence microscope IX73 model (Olympus) with an ultraviolet filter.

### 2.5. Scratch Assay

Scratch assay was performed for detecting the effect of rhodomyrtone on SW1353 cell migration, as described in the previous report [45]. Briefly, cells were grown to 90% confluence in 6-well plates and the wound areas were created with a sterile pipette tip. The cellular debris was washed with PBS, and cells in each well were treated with or without rhodomyrtone (0–3 *μ*g/mL) for 24 h. The wound closure was observed and captured with a phase-contrast inverted microscope (Olympus, Tokyo, Japan). The wound area was measured by NIH ImageJ software, version 1.46r. The experiments were repeated three times.

### 2.6. Transwell Migration Assay

The antimigration potential of rhodomyrtone was assayed by the transwell chamber in 24-well plates. Cells were treated with or without rhodomyrtone (0–3 *μ*g/mL) for 24 h. After incubation, the migrated cells were fixed and stained. The percentage of the migratory cells for each treatment was calculated as previously described [43].

### 2.7. Cell Invasion Assay

The ability of cancer cells to invade through the membrane coated with 30 *μ*g of Matrigel (Corning incorporated, Bedford, MA, USA) was determined by the Boyden chamber assay as previously described [43]. Briefly, SW1353 cells were pretreated with or without rhodomyrtone (0–3 *μ*g/mL) for 24 h. Cells in serum-free medium were plated onto the upper side of the chamber, while the serum-containing medium (10% FBS) was added into the lower side of the chamber. After 24 h of incubation, the invaded cells were fixed, stained, and photographed under an inverted microscope. The data are presented as percentage of cell invasion from five random fields. The experiments were repeated three times and each experiment was carried out in duplicate.

### 2.8. Cell Adhesion Assay

The adhesion ability of SW1353 cells on the Matrigel-coated 96-well plate was performed according to the previous method [43]. Percentage (%) of cell adhesion was calculated by comparing with the untreated control. The experiments were carried out in triplicate and three independent experiments were performed.

### 2.9. Gelatin Zymography

Gelatin zymography was performed for determining the effect of rhodomyrtone on MMP-2/9 activities in culture media. The conditioned media were collected and subjected to gelatin containing SDS-PAGE (0.1% gelatin). The gelatinolytic activity was analyzed as in our previous research [43].

### 2.10. Western Blot Analysis

The Western blotting analysis was performed to investigate the metastasis-related proteins. Proteins were mixed with sample buffer and boiled for 10 min. Denatured proteins were separated in the 8–12% SDS-PAGE and blotted onto a PVDF membrane (Millipore Corporation, Billerica, MA, USA). After that, the membranes were immunoblotted with an appropriate dilution of the specific primary antibody, followed by incubation with the appropriate secondary antibody conjugated with horseradish peroxidase (Cell Signaling Technology, Danvers, MA). The protein bands were detected by using Immobilon™ Western Chemiluminescent HRP Substrate (Merck KGA, Darmstadt, DE) and exposed to Chemiluminescent Imaging System (GeneGenome Gel Documentation, Synoptics Ltd., Cambridge, UK). The relative density was quantified as in our previous research [43].

### 2.11. Statistical Analysis

Data were reported as mean ± standard deviation (SD) of three independent experiments. Statistical significance was analyzed by a one-way ANOVA test. The differences between treatment groups and the untreated groups were considered statistically significant at *p* < 0.05. SPSS 17.0 software was carried out for statistical analyses.

## 3. Results and Discussion

### 3.1. Rhodomyrtone at Low Concentrations Did Not Affect Cell Viability in SW1353 Cells

**Figure 1:**
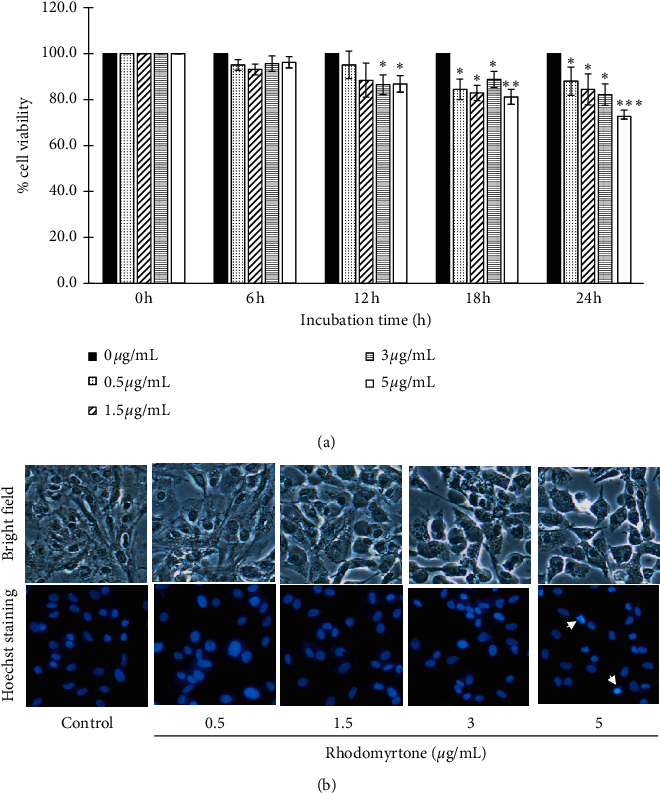
Effect of SW1353 cell viability by rhodomyrtone. (a) Cell viability was analyzed by MTT assay for 24 h. Rhodomyrtone inhibited SW1353 cell growth in a time- and concentration-dependent manner. (b) Nuclear morphology changes were detected by Hoeschst 33342 staining. Rhodomyrtone at 0–3 *μ*g/mL (cell viability >80%) was selected for subsequent experiments. The arrows indicate apoptotic cells. Data were expressed as mean ± standard deviation (SD) from three independent experiments.

Our previous study demonstrated that rhodomyrtone inhibited cell growth and induced apoptosis in skin cancer cells [44]. In this study, we investigated whether rhodomyrtone suppressed cell viability in human chondrosarcoma SW1353 cells. MTT assay was performed to determine the cell viability and cell proliferation effect of rhodomyrtone on SW1353 cells.  Figure 1(a) shows that rhodomyrtone suppressed SW1353 cell viability in a dose- and time-dependent fashion. Rhodomyrtone reduced cell viability of SW1353 cells at the high concentration (>3 *μ*g/mL) but at the lower concentration (0–3 *μ*g/mL) cell viability was >80%. Furthermore, we investigated the nuclear morphological changes of SW1353 cells after treatment with rhodomyrtone (0–5 *μ*g/mL).  Figure 1(b) shows that no apoptotic cells and nuclear morphological changes were detected after treatment with rhodomyrtone at concentration ranges 0–3 *μ*g/mL. Likewise, our previous study found that rhodomyrtone could inhibit A431 cell proliferation at high concentrations while at the lower concentration it did not [44]. Therefore, rhodomyrtone at concentration range (0–3 *μ*g/mL) was selected for subsequent experiments. This result indicates that rhodomyrtone at higher concentrations inhibited cell viability, but at the concentrations ranging from 0–3 *μ*g/mL it did not.

### 3.2. Rhodomyrtone at Subcytotoxic Concentrations Inhibited SW1353 Cell Migration

**Figure 2:**
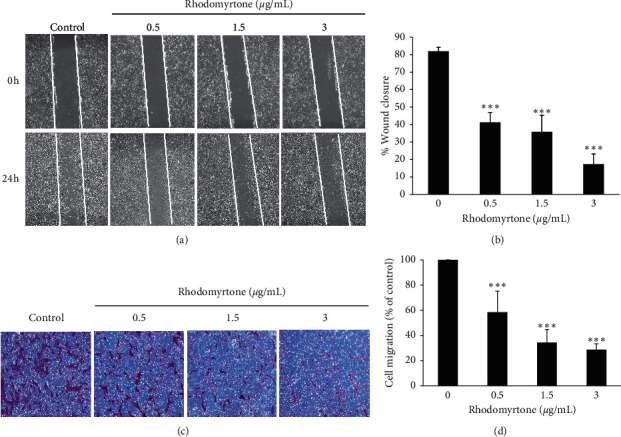
Antimigration of rhodomyrtone was determined by wound healing and transwell chamber assay. (a) The wound areas were created by a sterile micropipette tip and treated with or without the subcytotoxic concentrations of rhodomyrtone for 24 h. Cell migration of SW1353 cells was captured under a 40x magnification of the microscope. (b) The wound closure was analyzed by measuring the area between the edges (white lines indicated the wound edge). (c) Rhodomyrtone also significantly suppressed cell migration in a dose-dependent manner as detected with the transwell chamber. (d) Quantitative analysis of the migratory cells was calculated by NIH ImageJ. Data represent mean ± standard deviation (SD) from three independent experiments. ^*∗∗∗*^*p* < 0.001 vs. untreated control.

Cancer metastasis is the predominant cause of cancer-related death. Cell migration, invasion, and adhesion are key steps in tumor progression [6]. Disruption of any metastatic steps is a target for preventing the development of cancer metastases. In our previous study, rhodomyrtone has been reported to suppress skin cancer metastasis by inhibiting cell migration [43]. Thus, we determined the effect of rhodomyrtone on cancer cell migration in SW1353 cells. Wound healing and the transwell chamber assay were used to investigate the antimigration effect of rhodomyrtone in SW1353 cells. Rhodomyrtone has been shown to significantly reduce cell migration to the wound area after treatment with rhodomyrtone at the subcytotoxic concentrations (0.5, 1.5, and 3 *μ*g/mL) as shown in Figures 2(a) and 2(b). Cell migration rate was reduced to 41.4 ± 6%, 35.8 ± 9%, and 17.3 ± 6%, respectively. To confirm the antimigration potential of rhodomyrtone in SW1353 cells, a transwell chamber assay was performed. The result showed cell migration was significantly suppressed by rhodomyrtone in a concentration-dependent manner as shown in Figures 2(c) and 2(d). The inhibition of SW1353 cell migration by rhodomyrtone was independent of the direct cytotoxic effects on cancer cells.

### 3.3. Rhodomyrtone at the Subcytotoxic Concentrations Inhibited SW1353 Cell Invasion and Adhesion

**Figure 3:**
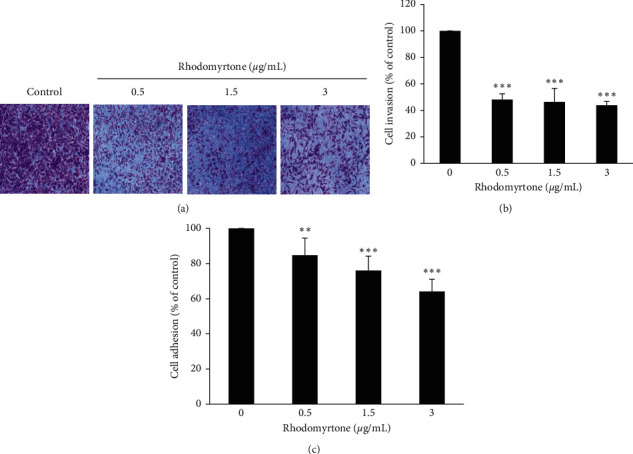
Cell invasion and adhesion assay. (a) Anti-invasion activity of rhodomyrtone was analyzed by the Matrigel-coated Boyden chamber assay. The cells invaded to the lower side of the chamber were stained and photographed under a 40x magnification of the microscope. (b) Percentage of invaded cells showed significant decrease after treatment with rhodomyrtone in a concentration-dependent manner. (c) Cells adhesion ability of treated cells was markedly inhibited after treatment with rhodomyrtone. The values are presented as mean ± standard deviation (SD) from three independent experiments. ^*∗∗*^*p* < 0.01 and ^*∗∗∗*^*p* < 0.001 vs. untreated control.

To explore the effect of rhodomyrtone on cancer cell metastasis in SW1353 cell, we investigated the inhibition of SW1353 cell invasion by rhodomyrtone using Matrigel-coated Boyden chamber assay. Figures 3(a) and 3(b) show that rhodomyrtone reduced the SW1353 cell invasion in a concentration-dependent manner (^*∗∗∗*^*p* < 0.001). The percentage of invaded cells was 48.2 ± 4.4%, 46.4 ± 10.1%, and 43.9 ± 2.9% when treated with 0.5, 1.5, and 3 *μ*g/mL of rhodomyrtone, respectively. Also, the inhibitory effect of rhodomyrtone on cell adhesion of SW1353 cells was performed by MTT assay. The result demonstrated that cell adhesion ability of SW1353 cells to Matrigel-coated wells could reduce at 84.8 ± 9.8%, 76.0 ± 8.2%, and 64.0 ± 7.1% after treatment by 0.5, 1.5, and 3 *μ*g/mL of rhodomyrtone (Figure 3(c)), respectively. These results indicated that rhodomyrtone inhibits chondrosarcoma cells metastasis without apparent cytotoxicity. Likewise, our previous study has also reported that rhodomyrtone at the nontoxic concentrations exhibited strong inhibition on human skin cancer cell metastasis by reducing cell migration, cell invasion, and cell adhesion ability [43].

### 3.4. Rhodomyrtone at the Subcytotoxic Concentrations Inhibited the Expression and Activity of MMP-2 and MMP-9

**Figure 4:**
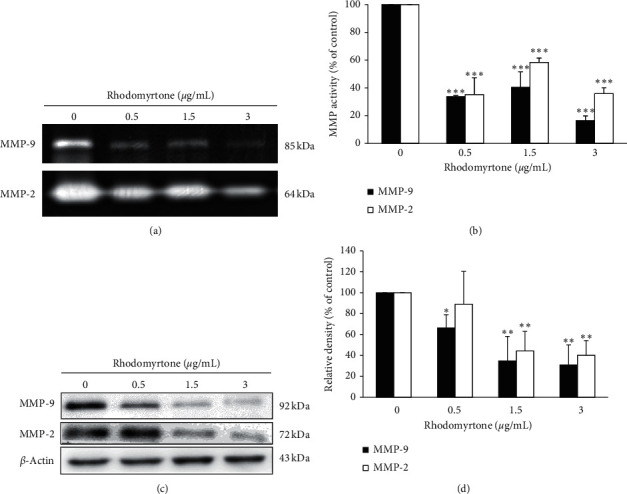
Effect of rhodomyrtone on MMP-2 and MMP-9 activities and protein expression. (a) Photograph presented the gelatinolytic activity of MMP-2 and MMP-9. (b) Quantitative analysis of MMP-2 and MMP-9 activities was calculated using NIH ImageJ. (c) Expression of MMP-2 and MMP-9 proteins was detected by using the specific antibodies. (d) Protein levels of MMP-2 and MMP-9 were significantly suppressed by rhodomyrtone in a concentration-dependent manner. Data are presented as mean ± standard deviation (SD) from three independent experiments. ^*∗*^*p* < 0.05, ^*∗∗*^*p* < 0.01, ^*∗∗∗*^*p* < 0.001 vs. untreated control.

Previous reports showed MMP-2 and MMP-9 expression was correlated with cancer invasion and the upregulation of MMPs was observed in invasive cancer cells [46–48]. The inhibition of MMP-2 and MMP-9 enzyme activity and protein expression has been shown to inhibit cancer cell migration and invasion in many types of tumor cells [49–52]. In this study, we investigated the expression and activity of MMP-2 and MMP-9 after treatment with rhodomyrtone at low concentrations. Gelatin zymography was performed to determine the activity of MMP-2 and MMP-9. The result demonstrated that rhodomyrtone significantly reduced the activity of MMP-2 and MMP-9 in a concentration-dependent manner as shown in Figures 4(a) and 4(b). The protein expression of MMP-2 and MMP-9 was determined by Western blot analysis. The result showed MMP-2 and MMP-9 protein expression was significantly suppressed by rhodomyrtone as compared to the untreated control as shown in Figures 4(c) and 4(d). These results revealed that the rhodomyrtone inhibited both MMP-2 and MMP-9 activities and protein expression in SW1353 cells. Thus, inhibition of MMPs activities and protein expression is the target for preventing cancer metastases. This is consistent with previous reports, showing that resveratrol attenuated MMP-9 and MMP-2 regulated differentiation of HTB94 cells [52]. Some studies demonstrated that curcumin and curcumin derivative inhibited cancer cell invasion through the downregulation of MMPs in human A549 lung cancer cells [53], MDA-MB-231 human breast cancer cells [54], MCF-7 cells [55], and hepatocellular carcinoma [56].

### 3.5. Rhodomyrtone at the Subcytotoxic Concentrations Induced the Expression Endogenous Inhibitor of MMP-2 and MMP-9

**Figure 5:**
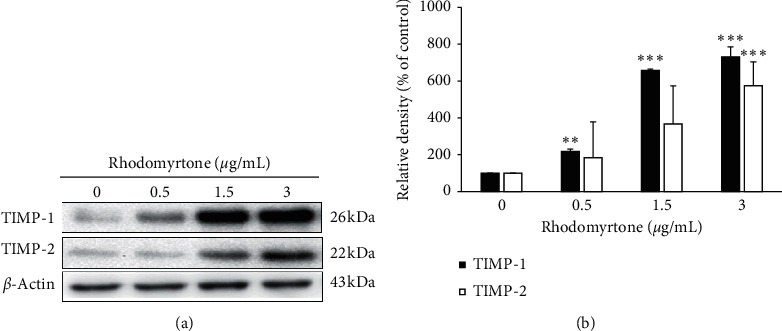
Western blot analysis of TIMPs protein level in SW1353 cells. (a) Expression of TIMP-1 and TIMP-2 protein was detected. (b) Rhodomyrtone significantly increased the expression of TIMP-1 and TIMP-2 protein levels. Data are presented as mean ± standard deviation (SD) from three independent experiments. ^∗^*p* < 0.05, ^*∗∗*^*p* < 0.01, ^*∗∗∗*^*p* < 0.001 vs. untreated control.

In this study, we found that the activities of MMP-2 and MMP-9 were inhibited by rhodomyrtone. The activities of MMPs are specifically inhibited by a group of tissue inhibitors of metalloproteinases (TIMPs); TIMP-1 and TIMP-2 have been known to interact with MMP-9 and MMP-2, respectively. Several studies reported that overproduction of TIMPs can reduce metastasis whereas a low level of TIMPs correlates with tumor progression [17, 57, 58]. In this research, the expression of TIMP-1 and TIMP-2 was analyzed by Western blot analysis. We found that rhodomyrtone significantly increased TIMP-1 and TIMP-2 protein expression in a concentration-dependent manner (Figures 5(a) and 5(b)). This is consistent with our previous study which showed that rhodomyrtone reduced A431 cell metastasis by suppressing MMP-2/9 activities and increasing the expression of TIMP-1 and TIMP-2 [43]. Similarly, Ferrari and colleagues showed that the upregulation of TIMP-1 by genipin could inhibit MMP-2 activity and suppressed the metastasis of HepG2 and MHCC97 L cells [57]. Likewise, the inhibition of A549 cell metastasis by increasing TIMP-2 expression [58]. This result indicated that the enzyme activities of MMP-2 and MMP-9 were inhibited by their endogenous inhibitors.

### 3.6. Rhodomyrtone at the Subtoxic Concentrations Downregulated Integrin *αv*, Integrin *β*3, FAK, AKT, and Rho GTPase Signaling Pathway

**Figure 6:**
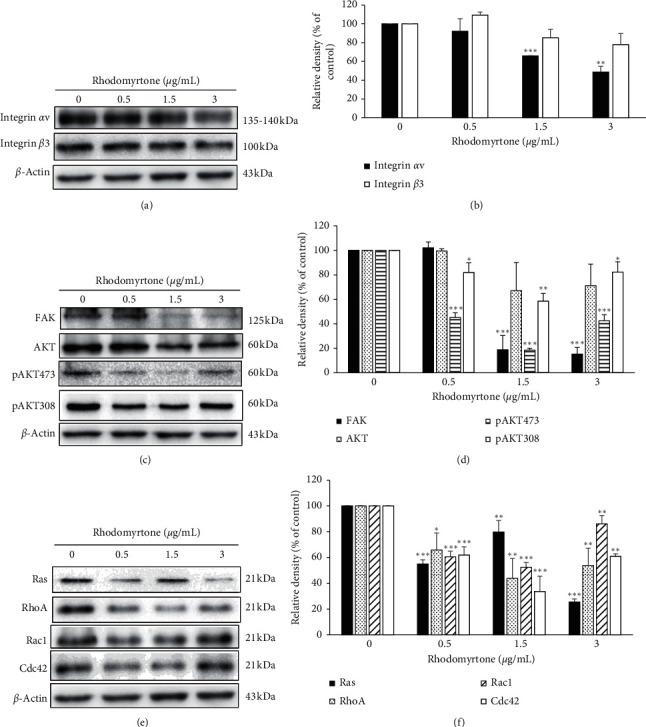
Western blot analysis of integrin *αv*, integrin *β*3 protein levels, and the effector proteins. (a) Western blotting analysis of *αvβ*3 integrin detected by using specific antibodies. *β*-Actin was used for equal loading. (b) Relative density of proteins was significantly inhibited after treatment with rhodomyrtone. (c) FAK, AKT, pAKT (Ser473), and pAKT (Thr308) were detected by using specific antibodies and *β*-actin is used as the loading control. (d) The relative density of proteins was significantly inhibited after treatment with rhodomyrtone in a concentration-dependent manner. (e) Specific antibodies were used for detecting Ras, RhoA, Rac1, and cdc42. *β*-Actin was used for equal loading. (f) The relative density of proteins was significantly inhibited after treatment with rhodomyrtone. Data are represented as mean ± standard deviation (SD) from three independent experiments. ^*∗*^^*∗*^*p* < 0.05, ^*∗∗*^*p* < 0.01, ^*∗∗∗*^*p* < 0.001 vs. the untreated control.

Western blot analysis was performed to clarify the molecular mechanism of rhodomyrtone on human chondrosarcoma cell metastasis. Integrins are cellular receptors that associate the extracellular matrix to intracellular signaling molecules and regulate many cellular processes, including cell migration, adhesion, survival, growth, and differentiation [59, 60]. Integrin *αvβ*3 has been reported to encourage cancer cell survival, migration, invasion, and angiogenesis in human chondrosarcoma [16]. Therefore, inhibition of integrin *αv* and integrin *β*3 expression is the good target for the metastasis treatment in human chondrosarcoma. We investigated whether rhodomyrtone could inhibit the expression of these metastasis-associated integrins. We found that rhodomyrtone at the subcytotoxic concentrations (0.5, 1.5, and 3 *μ*g/mL) significantly reduced integrin *αv* expression in a dose-dependent manner but not much on integrin *β*3 (Figure 6(a)). This finding indicates that rhodomyrtone inhibited SW1353 cell metastasis by modulating integrin *αv* and integrin *β*3. This is consistent with that of Wu and colleagues who showed berberine suppressed chondrosarcoma cell migration by inhibiting the *αvβ*3 integrin expression [61]. Moreover, we further evaluated the downstream effectors of integrin including focal adhesion kinase (FAK), AKT, and p-AKT and small Rho GTPases family. There are many reports indicating that integrins initiate transmembrane signaling through activation of FAK and activate the downstream effectors such as AKT and Rho GTPases families for cancer cell migration [8, 17–19]. Rho GTPases family including Ras, RhoA, Rac1, and Cdc42 are well known to regulate cytoskeleton remodeling, cell migration, malignant transformation, cell polarity, invasion, and metastasis [20]. Ras and Rac-1 regulate lamellipodia formation, Cdc42 stimulates plasma membrane protrusion filopodia, and RhoA promotes stress fiber formation [19, 55, 62, 63]. Western blot analysis showed that rhodomyrtone significantly suppressed FAK expression and the downstream effectors including total AKT, phosphorylated AKT, Ras, RhoA, Rac1, and Cdc42 in SW1353 cell (Figures 6(c) and 6(f)).

Consistent with the effect of artonin E on migration and invasion inhibition of lung cancer cells via suppression of activated FAK, activated AKT, and CDC42 [62], *Coptidis Rhizoma* inhibits hepatic carcinoma cell migration by inactivation of Rho signaling [64]. Likewise, inhibition of Rac1 in MDA-MB-231 cells was shown to inhibit lamellipodia formation and, subsequently, cellular migration [65]. These results suggested that rhodomyrtone inhibited cell migration by significantly suppressing integrin *αv*, but not much on integrin *β*3. The downstream migratory proteins of FAK, AKT, and Rho GTPase signaling pathways were also suppressed by rhodomyrtone.

### 3.7. Rhodomyrtone at the Subtoxic Concentrations Suppressed the Expression of ERK/JNK and Induced the Expression of p38 Signaling Pathway

**Figure 7:**
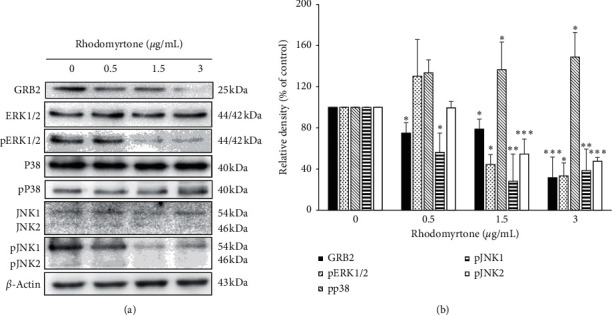
Western blot analysis of the protein level of GRB2 and MAPK pathway in SW1353 cells. (a) Expression of GRB2 and MAPK protein members was determined by Western blot analysis after treatment with or without rhodomyrtone and *β*-Actin is used as a loading control. (b) Relative density of proteins was significantly inhibited after treatment with rhodomyrtone. Data are presented as mean ± standard deviation (SD) from three independent experiments. ^∗^*p* < 0.05, ^*∗∗*^*p* < 0.01, ^*∗∗∗*^*p* < 0.001 vs. the untreated control.

In this study, we showed MMP-2 and MMP-9 expression was suppressed by rhodomyrtone at the subcytotoxic concentrations. MMP-2 and MMP-9 are the important proteases involved in cancer cell migration, spreading, invasion, and metastasis [30]. They are highly expressed in various malignant tumors. The activation of these enzymes enables the degradation of ECM by cancer cells allowing their access to the vasculature as well as their migration and invasion into the target organ and development of cancer metastasis [31, 33]. Mitogen-activated protein kinase (MAPK) signaling pathway especially has been reported to be involved in regulation of MMP-2 and MMP-9 expression [16, 66–69]. Reduction of MAPK protein members may have the potential to interrupt cancer cell migration, invasion, and progression. Here, we investigated the effect of rhodomyrtone on MAPK protein members in SW1353 cells. The results showed that GRB2, phosphorylated ERK1/2, and JNK1/2 were significantly inhibited after treatment with rhodomyrtone in a concentration-dependent manner (Figure 7). Many previous studies showed that the expression of MMPs such as MMP-1, MMP-2, MMP-9, and MMP-13 in human chondrosarcoma is regulated by integrin *αvβ*3/FAK/ERK pathway [17, 70–72]. Likewise, the previous report demonstrated that expression of MMP-2 and MMP-9 in cancer cells was regulated by JNK/MAPK pathway [73]. Our previous study showed rhodomyrtone inhibited skin cancer cell metastasis by suppression of MMP-2/9 expression through inhibiting ERK1/2 and p38 signaling pathway [43]. Moreover, we found that rhodomyrtone induced expression of phosphorylated p38. Activation of p38 MAPK signaling has been reported to be involved in the overexpression of TIMP-1, the endogenous inhibitor of MMP-2, and it may be responsible for MMP-2 inhibition [56]. These findings suggest that rhodomyrtone at the subcytotoxic concentrations inhibited human chondrosarcoma cell invasion by suppressing MMP-2 and MMP-9 expression via ERK and JNK signal inhibition.

## 4. Conclusions

**Figure 8:**
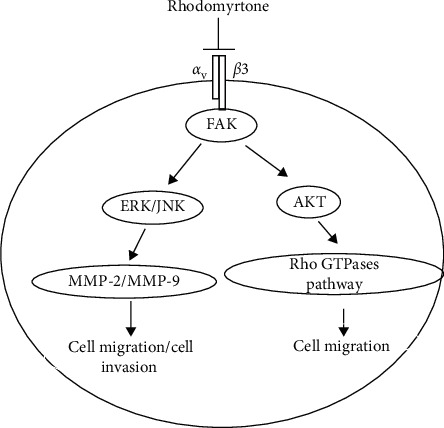
Rhodomyrtone inhibited SW1353 cell metastasis by suppressing integrin *αvβ*3/FAK/AKT/small Rho GTPases pathway as well as downregulation of MMP-2/9 via ERK and JNK signaling pathway inhibition.

Our present study demonstrated that rhodomyrtone could inhibit SW1353 cell motility, migration, invasion, and adhesion by suppressing integrin *αvβ*3/FAK/AKT/small Rho GTPases pathway as well as downregulation of MMP-2/9 via ERK and JNK signaling pathway inhibition (Figure 8). These findings indicate that rhodomyrtone is a new antimetastasis agent for the treatment of cancer metastasis. In vivo studies need to be performed in the next phase to determine the therapeutic efficacy.

## Data Availability

The data and materials used in the study are available from authors upon reasonable request.
